# Future Leaders to Watch – Andrea Paterlini

**DOI:** 10.1242/bio.057356

**Published:** 2020-11-19

## Abstract

Future Leaders to Watch is a series of interviews with the first authors of a selection of papers published in Biology Open, helping early-career researchers promote themselves alongside their papers. Andrea Paterlini is author of ‘Uncharted routes: exploring the relevance of auxin movement via plasmodesmata’, published in BiO. Andrea is a PhD student in the groups of Yka Helariutta and Ottoline Leyser at the Sainsbury Laboratory, University of Cambridge, investigating the structure, function and regulation of plasmodesmata, the small pores that connect plant cells.


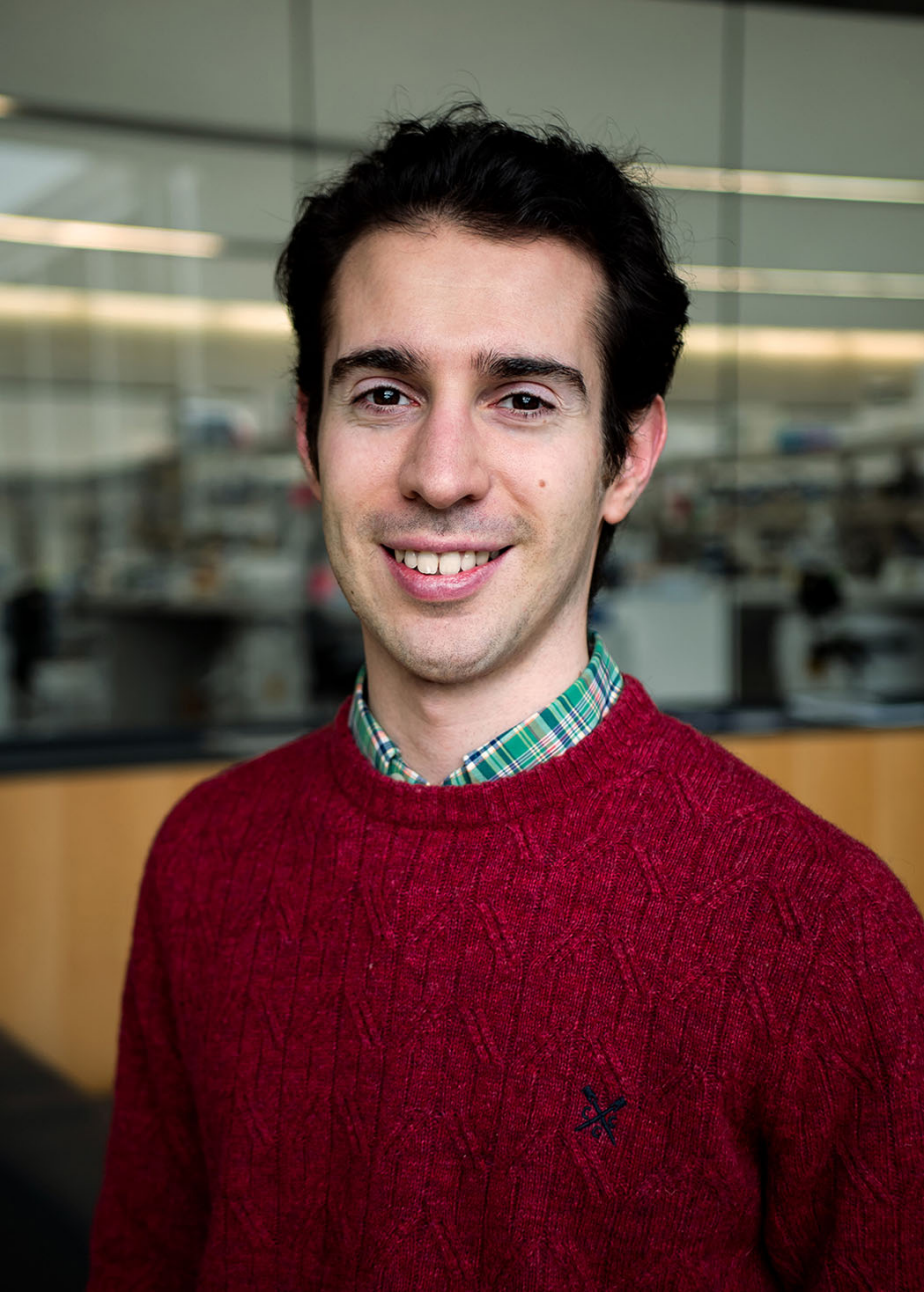


**Andrea Paterlini**

**What is your scientific background and the story of how you got to where you are today?**

When I started my undergraduate degree at the University of Edinburgh, in 2012, I was initially registered on a biology programme focused on infectious diseases. However, early on, I became fascinated with plant science and with plasmodesmata, the microscopic channels enabling transport and communication between plant cells. The *Gatsby Plant Science Summer School*, at the end of my second year, was also instrumental in my decision to switch subject. In parallel to my courses I got to work with Karl Oparka, whose enthusiasm for plasmodesmata rubbed off on me. In his group we developed imaging approaches for cell-to-cell and long-distance trafficking. For my PhD, in 2016, I moved to the Sainsbury Laboratory at the University of Cambridge. I joined the groups of Yka Helariutta and Ottoline Leyser to look at plasmodesmata in the context of plant development. I elucidated plasmodesmata ultra-structure upon lipid perturbations and developed computational tools to study the plasmodesmata environment. My training and professional development was generously supported by the Gatsby Charitable Foundation. I have now just submitted my thesis and I am preparing some new manuscripts.

“I became fascinated with plant science and with plasmodesmata, the microscopic channels enabling transport and communication between plant cells.”

**What is the most important take-home message of your review?**

I try to stress that cell-cell movement of the hormone auxin, an extremely important substance for plant growth and development, via plasmodesmata connections, is extensive, complex, likely regulated and with biological roles in various developmental contexts.

**What has surprised you the most while researching this review?**

I approached this review thinking that auxin movement via plasmodesmata was itself a contentious idea and that it was only recently gaining traction. This was rather incorrect. Albeit this type of movement (compared to auxin transport via active transporter proteins embedded in the membranes of cells) was rarely emphasized in the literature, there was no reason to believe that it *was not* taking place. Some authors looking at auxin transport in the early days had indeed explicitly considered it. Graeme Mitchinson, who sadly passed away two years ago and I was lucky enough to spend time with in Cambridge, was among them. He had been a pioneer in this regard too. The central debate on auxin movement via plasmodesmata, as Ottoline Leyser pointed out to me in a conversation, was if such transport had *biological relevance* (rather than being a side effect of the auxin molecule fitting through plasmodesmata apertures). The recent publications I highlight in the review argue for a functional role of this transport.

**What do you feel is the most important question that needs to be answered to move the field forward?**

While the importance of auxin transport via plasmodesmata has been displayed in a few specific cases, additional examples are needed to establish how broad this relevance is. Revisiting developmental processes where auxin fluxes play central roles could be a valuable starting point. Integrating the many routes of auxin movement, their reciprocal feedbacks and their regulations, will be exciting challenges in coming years.

**Figure:**
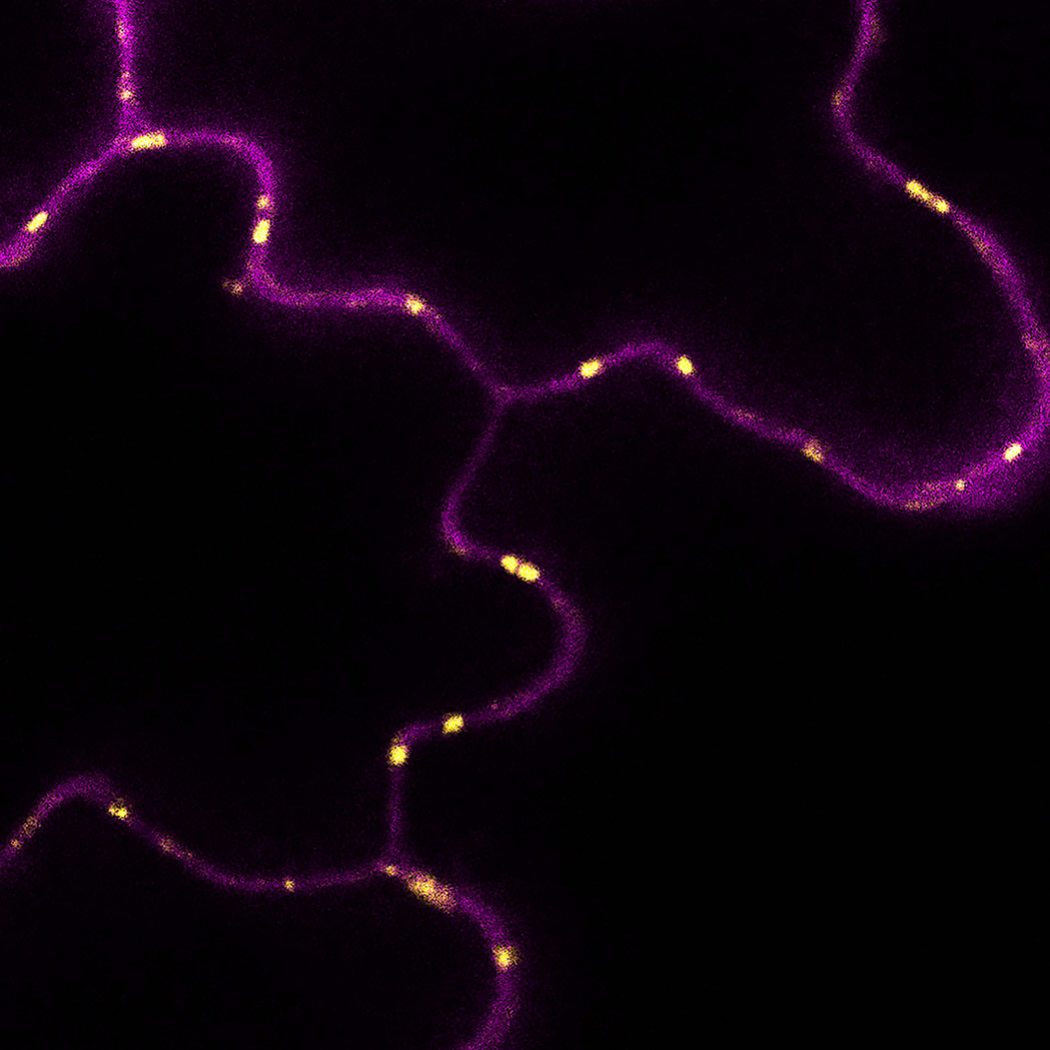
**Protein marker highlighting plasmodesmata (false coloured in yellow) connecting cells of the leaf epidermis (false coloured in magenta).**

**What changes do you think could improve the professional lives of early-career researchers?**

Early-career researchers can benefit from two things: opportunities to share their insights and ideas and the courage to take on those opportunities. While the latter is upon us the former should be more widely provided. I want to commend *Biology Open* for establishing the Future Leaders Reviews initiative, which is a great example of removing conventional entry barriers. The metrics early-career researchers are evaluated upon (and the idea of research contributions in general) also need to be broadened beyond publications as we also teach future generations of scientists, engage with the public and follow personal vocations. I want to mention the *Royal Society* initiative ‘Resume for scientists’, which addresses many of these aspects. Establishing networks of early-career researchers is also essential to shape a collaborative future research community.

“Early-career researchers can benefit from two things: opportunities to share their insights and ideas and the courage to take on those opportunities.”

**What's next for you?**

In November I will start a Postdoctoral position at the Laboratory of Membrane Biogenesis (Bordeaux – France) in the group of Emmanuelle Bayer. There I will continue to probe the secrets of plasmodesmata, this time focusing on how lipid changes might accompany (and perhaps be required for) structural deformations of these pores. New challenges and exciting times await me, *allons-y* (let's go)!
